# Phenotypically Dormant and Immature Leukaemia Cells Display Increased Ribosomal Protein S6 Phosphorylation

**DOI:** 10.1371/journal.pone.0151480

**Published:** 2016-03-17

**Authors:** Monica Pallis, Tamsin Harvey, Nigel Russell

**Affiliations:** 1 Clinical Haematology, Nottingham University Hospitals, Nottingham, United Kingdom; 2 Department of Haematology, University of Nottingham, Nottingham, United Kingdom; Queen's University Belfast, UNITED KINGDOM

## Abstract

Mechanistic/mammalian target of rapamycin (mTOR) activity drives a number of key metabolic processes including growth and protein synthesis. Inhibition of the mTOR pathway promotes cellular dormancy. Since cells from patients with acute myeloid leukaemia (AML) can be phenotypically dormant (quiescent), we examined biomarkers of their mTOR pathway activity concurrently with Ki-67 and CD71 (indicators of cycling cells) by quantitative flow cytometry. Using antibodies to phosphorylated epitopes of mTOR (S2448) and its downstream targets ribosomal protein S6 (rpS6, S235/236) and 4E-BP1 (T36/45), we documented that these phosphorylations were negligible in lymphocytes, but evident in dormant as well as proliferating subsets of both mobilised normal stem cell harvest CD34+ cells and AML blasts. Although mTOR phosphorylation in AML blasts was lower than that of the normal CD34+ cells, p-4E-BP1 was 2.6-fold higher and p-rpS6 was 22-fold higher. Moreover, in contrast to 4E-BP1, rpS6 phosphorylation was higher in dormant than proliferating AML blasts, and was also higher in the immature CD34+CD38- blast subset. Data from the Cancer Genome Atlas show that rpS6 expression is associated with that of respiratory chain enzymes in AML. We conclude that phenotypic quiescence markers do not necessarily predict metabolic dormancy and that elevated rpS6 ser235/236 phosphorylation is characteristic of AML.

## Background

Tumour cell growth is driven by active biosynthetic and glycolytic pathways [1] fuelling interest in finding anti-cancer uses for drugs which interfere with these processes [2–5]. Mechanistic/mammalian target of rapamycin (mTOR) is an element of the mTORC1 signalling complex which drives energy generation, macromolecule synthesis and cell growth [6–8]. Constitutive activation of mTOR is commonly found in tumour cells, but in quiescent normal cells mTOR activity and biosynthetic pathways are suppressed [1, 5]. This may happen in an energy-rich and nutrient-replete environment, such as in the case of circulating lymphocytes [9, 10], or may be a homeostatic response to nutrient or energy depletion in which AMPK is activated and mTOR subsequently inactivated to promote conservation of essential cell functions [1, 4, 11]. What remains unclear in these scenarios is the behaviour of the dormant cancer cell. Reversible exit from the cell cycle into the quiescent, G0 state is well described in somatic cells, and is characterised by small size and low RNA and protein synthesis [12, 13]. The mitogenic factors driving malignant transformation might be thought not to permit a state of true (G0) quiescence in cancer cells [13]. Nevertheless, in acute myeloid leukaemia, dormant (apparently quiescent) cells which retain proliferative potential have been described [14, 15]. A high proportion of circulating and bone marrow blasts in AML also have phenotypic features of dormancy, as measured by lack of Ki-67 [16]. Ki -67 is expressed in all active phases of the cell cycle including G1[17].

Standard chemotherapy for AML tends to spare dormant leukaemia cells [16, 18], so it would be useful to characterise this subset in order to establish how best to target it. Do dormant leukaemia cells better resemble normal dormant cells or proliferating cancer cells? To further our understanding of Ki-67¯ leukaemia cells, particularly with regard to their metabolic activity and hence potential susceptibility to therapeutic inhibition of this activity, we have measured biomarkers of mTOR activation status in presentation samples, using flow cytometry. This technique has enabled us to examine mTOR activation concurrently with proliferation status at the single cell level. We have measured activation-related epitopes of mTOR, 4E-BP1 and ribosomal protein S6, in conjunction with Ki-67 or the transferrin receptor CD71 and maturation markers, in primary cells of pre-treatment samples from patients with AML. MTOR phosphorylation was measured at serine 2448. This phospho-epitope is lost when raptor is depleted, indicating its specificity for mTORC1 [19]. MTOR is phosphorylated at serine 2448 by p70S6 kinase: whereas the phosphorylation is not thought to be intrinsically “activating”, it can be used as an indicator of the level of mTOR signalling because p70S6 kinase activity is, in turn, mTOR-dependent [20, 21]. S6 kinase also phosphorylates ribosomal protein S6 (rpS6) [22]. Antibodies to ribosomal protein S6 (rpS6) phosphorylated at serine 235/236 have been optimised for flow cytometry, where they are well-established as biomarkers for mTORC1 activity [23, 24]. A second major target of mTOR is 4E-BP1, which is directly phosphorylated by mTOR at T36/T45 [25]. 4E-BPs control protein synthesis [5, 26] and mediate mTORC1-dependent cell proliferation [27].

Conditional deletion of raptor from AML cells has revealed that mTORC1 deficiency increases the proportion of undifferentiated and self-renewing haematopoietic cells and decreases the proportion of differentiated cells in vivo [28], suggesting that mTOR might be most activated in the context of maturation rather than self-renewal. This would have implications for mTOR as a therapeutic target, and we therefore also measured mTOR activity in undifferentiated (CD34+CD38-) AML cells and those with monocytoid maturation compared to blast cells.

## Materials and Methods

### Cells

AML patient samples were obtained with written, informed consent according to the protocol approved by National Research Ethics Service Committee East Midlands—Nottingham 1 and Nottingham University Hospitals NHS Trust. Cells from G-CSF-mobilised donor stem cell harvests (SCH) were obtained with written, informed consent according the protocol approved by National Research Ethics Service Committee East Midlands—Nottingham 2 and Nottingham University Hospitals NHS Trust. Cells were processed by standard methods to mononuclear cell preparations. The KG1a myeloid leukaemia cell line was obtained from the European Collection of Animal Cell Cultures (Salisbury, UK) and was maintained in RPMI 1640 medium with 10% foetal calf serum (FCS; First Link, Birmingham, UK) and 2mM L-glutamine. KG1a experiments were performed with cell lines in log phase and were authenticated by genetic fingerprinting.

### Antibody labelling

In order to allow metabolic activity to resume after processing, thawed or freshly isolated mononuclear cell preparations were rested for 90 minutes in medium consisting of 20% foetal calf serum (First Link) in RPMI. They were fixed using paraformaldehyde to a final 2% concentration. After 15 minutes’ incubation at room temperature (RT), neutralising buffer (1.7 M Tris base, 1.25 M Glycine, pH 9.1) was added at twice the volume of paraformaldehyde solution and the cells were incubated for a further 15 minutes. Cell membranes were permeabilized with intracellular staining buffer containing 0.1% saponin. Cells were labelled with Alexa Fluor 647-conjugated antibodies to mTOR p-serS2448 (BD#564242), 4E-BP1 p-thr36/45) (BD#560286) or rpS6 p-ser235/236 (CST#4851), and with antibodies to Ki-67 (BD556026) or CD71 (BD #555537). The phenotype of the cells was determined using CD45, CD34 and CD38 antibodies. In the stem cell harvest analysis CD38 was replaced by CD2. Labelling took place overnight at 4°C. Preliminary experiments indicated that the cell surface phenotype was not affected by this procedure, whilst sensitivity for the Ki-67 and mTOR pathway epitopes was improved compared to a short incubation (data not shown).

### Flow cytometry and standardisation

Fluorescence overlap was compensated and data were collected on a FACSCanto II flow cytometer (Becton Dickinson). Data were analysed with FACS Diva software (Becton Dickinson). Additional graphics were produced using Flowing software (www.flowingsoftware.com). In order to be able to compare expression levels obtained on different occasions, all mTOR, rpS6, 4E-BP1 and their isotype control mean fluorescence intensity values were converted to standardised values using standard curves obtained with Rainbow Calibration Beads (BD#559123), before subtracting control from test values. The resulting values termed standardised relative fluorescence intensity (SRFI) units were used throughout. In samples with <1% events in the CD34^+^CD38^-^ subset, this subset was not included, but otherwise sufficient events were counted (10,000–200,000) to allow a reliable coefficient of variation, as described previously [29].

#### Statistics

Comparisons between paired subsets were made using the Wilcoxon signed rank test and between blasts and normal CD34^+^ cells by Mann Whitney analysis, with SPSS software.

## Results

### 1. Methodology

**Fig 1:**
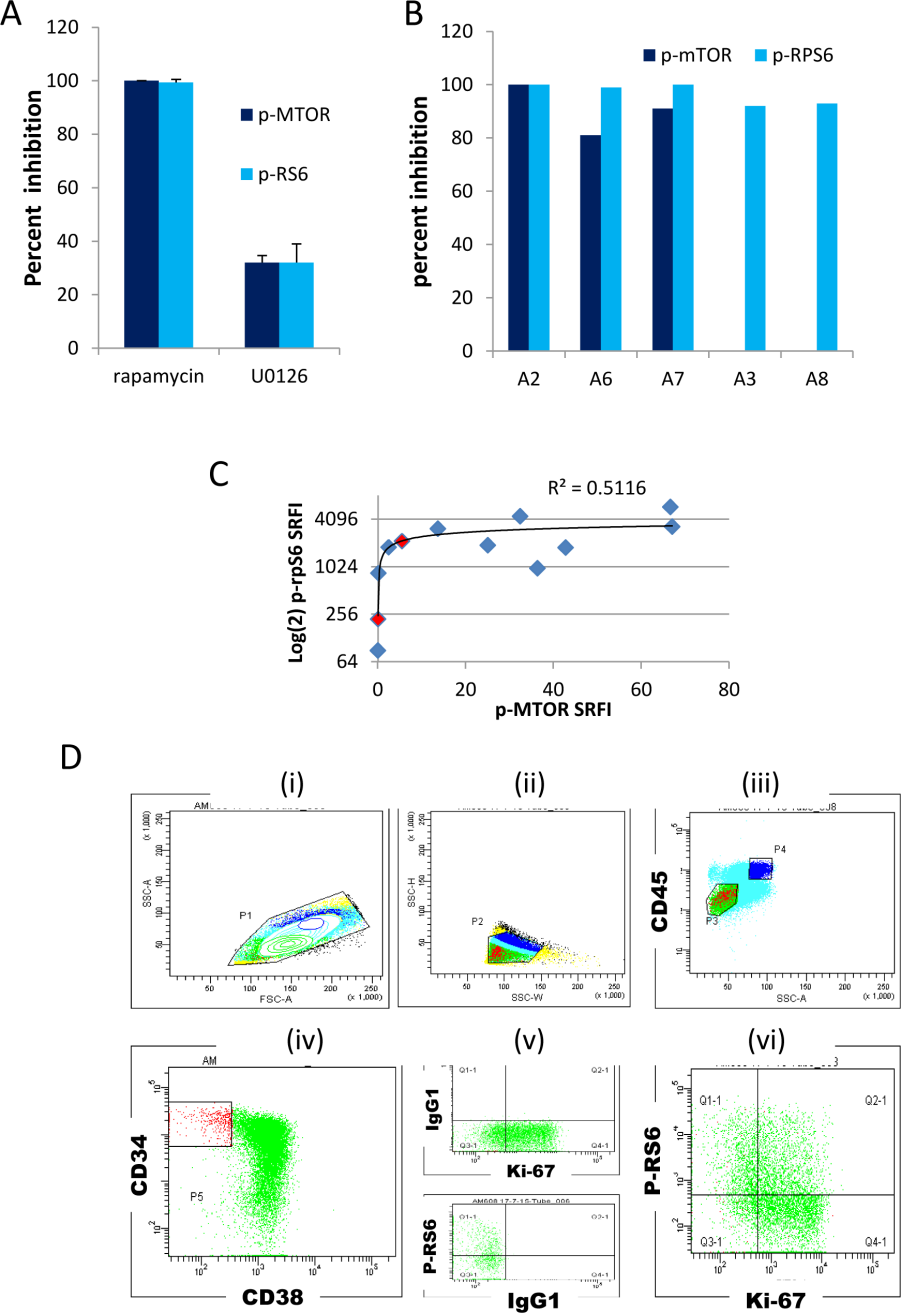
Methodology. (A) Inhibition of p-mTOR and p-rpS6 fluorescence in KG1a cells (mean + standard deviation of three independent assays) following a 90 minute incubation with 100nM rapamycin or 3 μM U0126. The percentage inhibition was established by also incubating cells with 100nM rapamycin + 3 μM U0126 to generate a functional fluorescence baseline. (B) Inhibition of -rpS6 and p-mTOR fluorescence in five primary samples following a 90 minute incubation with 100nM rapamycin. P-mTOR modulation was not measured in the two samples with a negligible basal signal (A3, A8). A fluorescence baseline was generated as for (A) above. (C) Scatterplot and trendline of p-rpS6 against p-mTOR in patient blasts, Spearman’s rho = 0.54, P = 0.05). SRFI (standardised relative fluorescence intensity) values were calculated as detailed in Materials and Methods. The two values shown in red correspond to samples A3 and A8 used in Fig 1B, such that taken together the plots illustrate that rpS6 phosphorylation is rapamycin-sensitive (plot 1B) despite the low measured values for mTORser2448 phosphorylation in these two samples (Plot 1C) and is thus p-rpS6 is a useful surrogate when the p-mTOR antibody signal is negligible. (D) Gating strategy; (i) and (ii) Scatter gates to exclude doublets and debris; (iii) CD45/side scatter gate to select leukaemic blasts (P3) or cells with monocytoid maturation (P4); (iv) gating of CD34^+^/CD38¯ blast subset; (v) Fluorescence minus one tube analysis to set accurate isotype control fluorescence for Ki-67 (FITC) and p-mTOR or p-rpS6 (APC) in P3-gated blasts; (vi) Ki-67 and antigen of interest co-expression in P3-gated blasts. Quadrants and fluorescence baselines were set using isotype controls. Mean APC fluorescence intensity for (Q1-1+Q3-1) dormant and (Q2-1 + Q4-1) cycling cells is recorded.

We measured mTOR phosphorylation at serine 2448 (hereafter termed p-mTOR), which is dependent on mTOR kinase activity [20]. We also measured ribosomal protein S6 phosphorylated at serine 235/236 (hereafter termed p-rpS6) [22, 30], a downstream target of mTORC1. Previous studies have shown p-rpS6 to be a sensitive marker for mTORC1 activation in flow cytometric studies [23, 24, 31]. However, although ERK activation can stimulate mTOR activity through TSC2 [32], rpS6 can be additionally phosphorylated at S235/236 in an mTORC1 independent/ERK dependent manner by p90S6 kinase [33]. To confirm the mTORC1 dependence of rpS6 S235/236 phosphorylation in AML cells, we first incubated cells with rapamycin with and without the MEK (ERK pathway) inhibitor U0126 in KG1a cells and measured p-mTOR and p-rpS6. At a concentration where U0126 induced 32% inhibition of both phospho-epitopes rapamycin inhibited both epitopes by 99%, confirming that MEK-mediated activation of rpS6 is upstream of mTOR in these cells and that p-rpS6 is a reasonable surrogate for rapamycin-sensitive mTORC1 activation (Fig 1A). In patient blasts, p-rpS6 and p-mTOR were also totally or mostly inhibited by rapamycin (80–100%, median 95%, Fig 1B). Fig 1C indicates a rationale for why p-rpS6, with a much stronger signal than p-mTOR, is a useful surrogate for mTORC1 activity. AML samples were selected to provide a wide range of cytogenetic and molecular abnormalities (Table 1). The gating strategy for comparing Ki-67 positive and negative blasts is indicated (Fig 1D). The use of CD45/side scatter (SSC) gating to distinguish blasts from lymphocytes and from leukaemia cells with cytoplasmic maturation and monocytoid differentiation (SSC^high^/CD45^high^) is well described in the literature [34–37].

**Table 1:** AML cases: demographics and phenotypes.

ID	NPM: 0 = wt, 1 = mut	FLT3: 0 = wt, 1 = ITD	Cytogenetics	age	FAB	% CD34+ blasts	% CD34+CD38- blasts	% ki-67+ blasts	% CD71+ blasts
A1	0	1	add5q, add11q	41	unknown	21	<1	42	ND
A2	0	0	11q23	56	M5	<1	<1	7	2
A3	0	1	normal	61	M4	12	6.0	37	42
A4	0	0	inv(16)	43	M4	37	7.1	81	76
A5	0	0	normal	50	M5	54	<1	41	ND
A6	unknown	0	inv(16)	65	M4e	14	6.0	57	35
A7	0	0	Normal	67	unknown	32	<1	51	66
A8	0	1	normal	20	M5	72	2.6	69	86
A9	0	0	normal	80	M1	97	8	37	ND
A10	unknown	1	failed	75	M0	50	10	72	ND
A11	0	0	t(8;21)	54	M2	74	7.8	38	ND
A12	1	0	abn(17p)	59	M2	1.5	<1	41	68
A13	1	1	normal	43	M1	28	1.4	34	84
A14	0	0	t(8;21)	41	M2	66	6	47	ND
A15	0	0	Complex	49	M1	<1	<1	72	ND
A16	1	0	Abn3q	72	M4	<1	<1	29	ND

ND = not done

### 2. The p-rpS6ser235/236 signal is amplified in AML blasts compared to mobilised normal CD34^+^ cells

**Fig 2:**
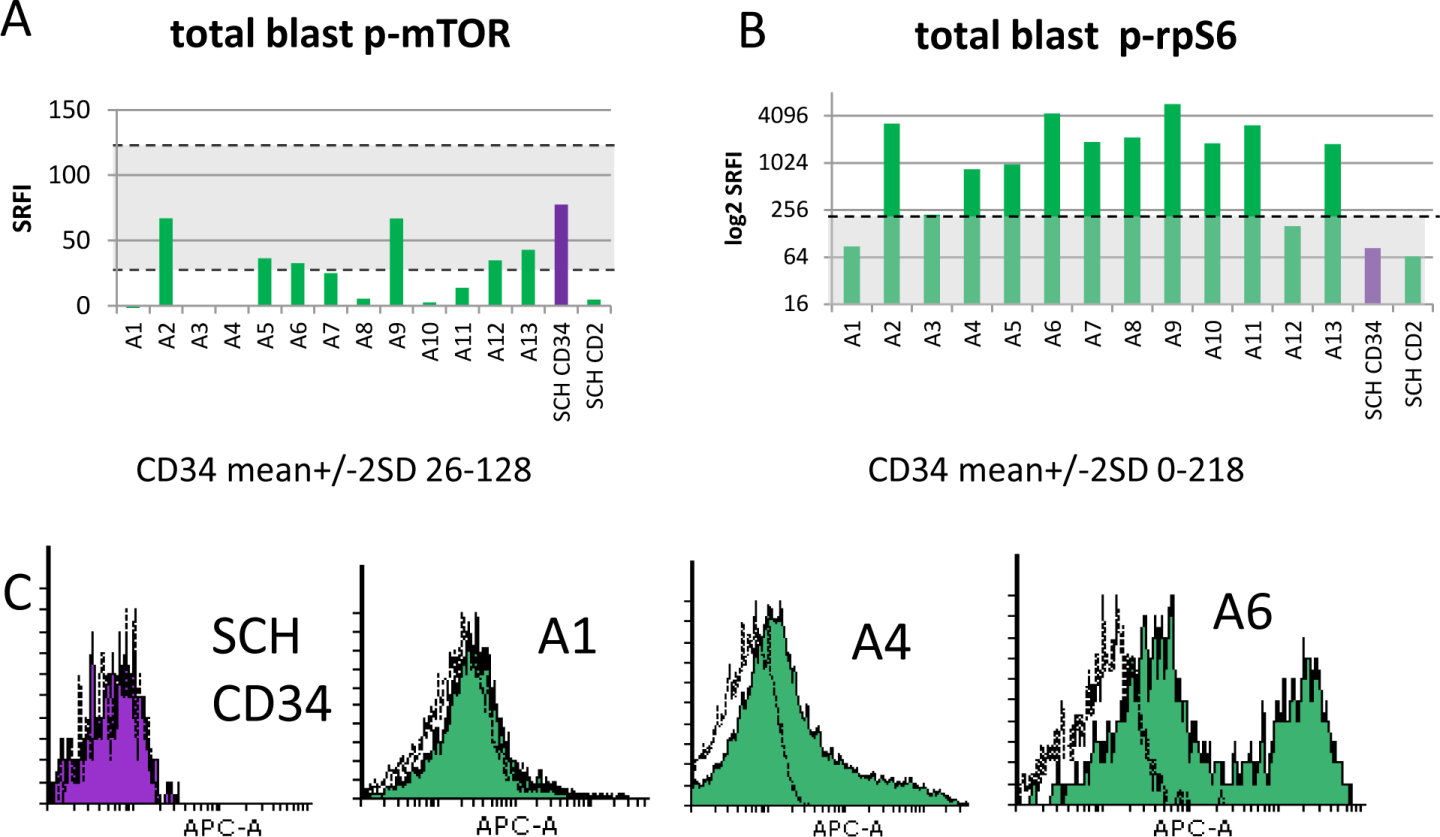
MTORser2488 and rpS6ser235/236 phosphorylation in AML blasts and normal CD34^+^ cells. Summary histograms for p-mTOR (A) and p-rpS6 (B) show standardised relative fluorescence intensity values (SRFI) for 13 individual primary AML samples and pooled data for five stem cell harvests (SCH). The latter were gated for subsets expressing CD34 or CD2. The shaded area indicates ± 2 standard deviations of the mean for the normal CD34^+^ cells. C. Flow cytometric distributions of p-rpS6 for one representative stem cell harvest (CD34^+^ gated cells) and three patient samples (P3 blast-gated cells–see Fig 1D). Flow cytometric histograms illustrate fluorescence signals from test antibody (shaded) compared to isotype control (unshaded).

Total p-mTOR-ser2448 expression was compared in blasts from presentation AML samples and mobilised SCH CD34^+^ and CD2^+^ cells. P-mTOR was negligible in the lymphocytes. Using a mean +/- 2 standard deviation range for the five stem cell harvest CD34^+^ subsets, we calculated that no AML sample had p-mTOR greater than the normal CD34^+^ cells (Fig 2A). In contrast, AML blast rpS6 phosphorylation was higher than that of CD34^+^SCH cells in 11/13 samples (Fig 2B, P = 0.002). Marked heterogeneity was noted both between and within patient samples (Fig 2C).

### 3. The mTOR-RPS6 pathway remains active in dormant AML cells

**Fig 3:**
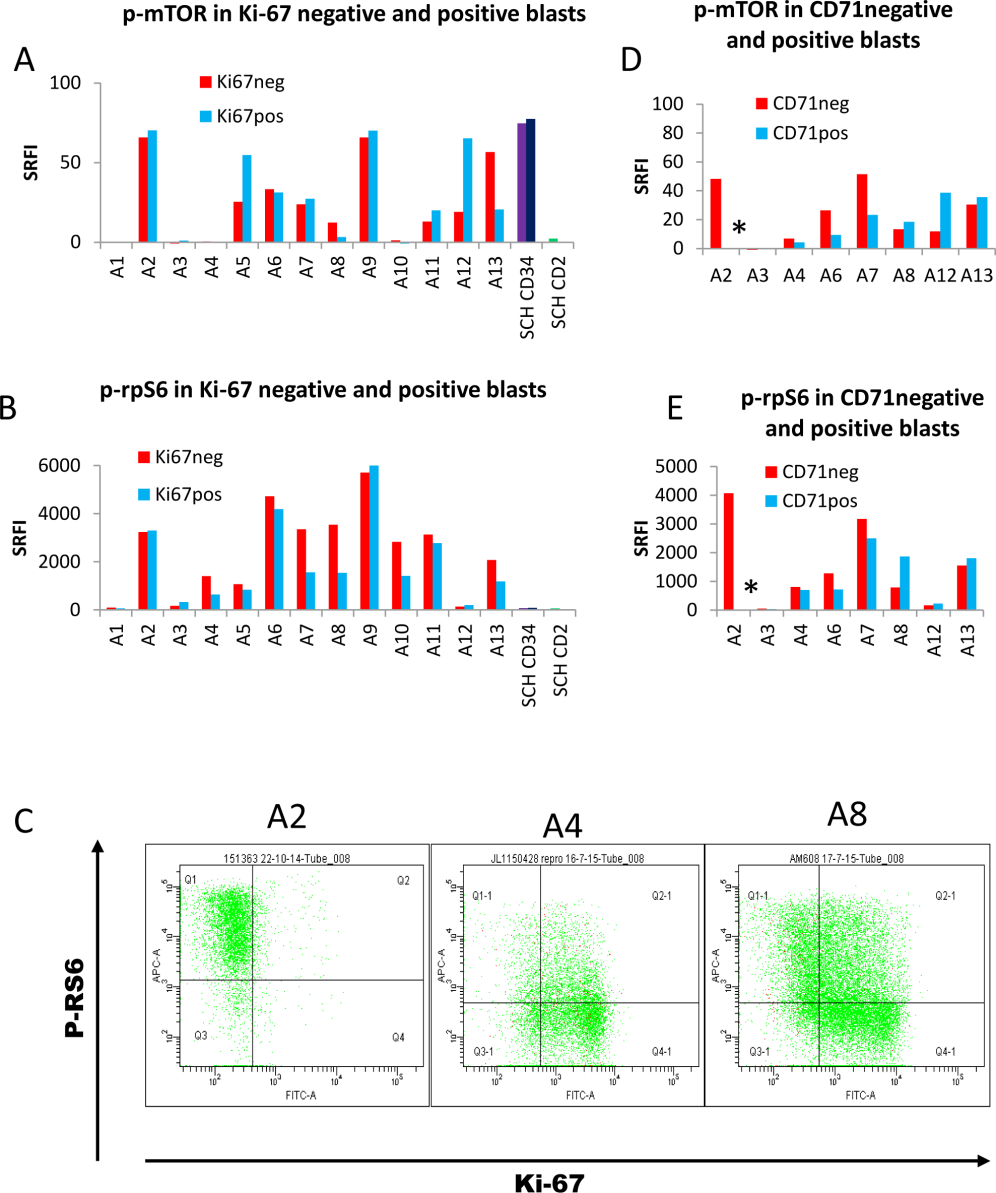
P-mTOR and p-rpS6 in dormant and proliferating cells. Summary histograms for p-mTOR (A,D), and p-rpS6 (B,E) show standardised relative fluorescence intensity unit values (SRFI) for Ki-67¯ and Ki67^+^ (A,B) and CD71¯ and CD71^+^ positive (D,E) blasts from 13 primary AML samples and five stem cell harvests. C. Flow cytometric distributions of p-rpS6 versus Ki-67 for three patient samples (P3 blast-gated cells). * = <1% CD71^+^ cells detected.

We reasoned that p-mTOR would be lowest in dormant cells and therefore compared Ki-67 positive and negative blasts. Contrary to expectation, we found no overall difference in mTOR activation between these subsets (Fig 3A). However, we also found 70% of normal SCH CD34^+^ cells to be Ki-67^+^, also with no difference in mTOR phosphorylation between the Ki-67¯ and Ki-67^+^ subsets, indicating that the Ki-67^-^ subset with active mTOR is not a leukaemia-specific phenotype. SCH CD2^+^ (lymphocyte subset) cells were 99% Ki-67^-^ and these had negligible p-mTOR, as expected [17]. Since rpS6 S235/236 phosphorylation is blocked by rapamycin, we also expected this to be low in dormant cells. However, p-rpS6 tended to be higher in Ki-67¯ than Ki-67^+^ subsets, and using Wilcoxon signed rank test, the difference was significant (Fig 3B, P = 0.03). Heterogeneity both between and within samples was marked (Fig 3C).

CD71, the transferrin receptor, is expressed on all proliferative cells, and its absence is characteristic of dormant populations such as resting lymphocytes [38]. In a previous publication, we showed that AML cells can express the transferrin receptor CD71 whilst remaining Ki-67^-^ [18] and therefore we wondered whether a CD71^+^ population might include a subset metabolically primed to enter G1 but without Ki-67 expression. No consistent pattern emerged from our analysis (Fig 3D and 3E). Notably, sample A2 expressed high levels of p-rpS6, although it had <1% CD71¯ blasts. However, in samples A8 and A13, in both of which expression of CD71 was high (see Table 1), the relative overexpression of p-rpS6 in the Ki-67¯ subset was reversed in the CD71¯ subset.

### 4. Ribosomal protein S6 is phosphorylated in CD34^+^CD38^-^ subsets and in cells with myeloid maturation

**Fig 4:**
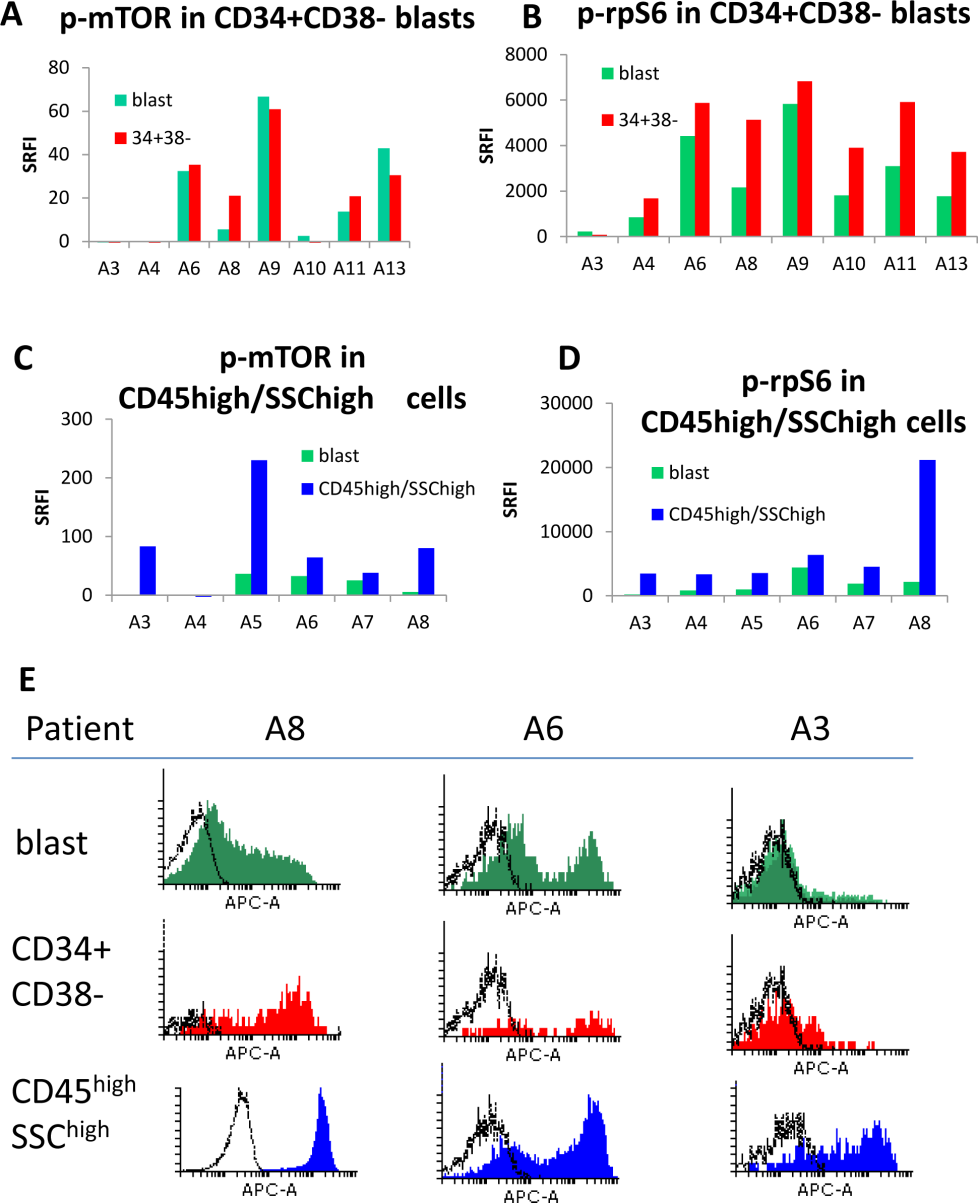
Subset analysis. Summary histograms of p-mTOR (A,C), and p-rpS6 (B,D) for (A,B) CD34^+^CD38^-^ and (C,D) CD45^high^/side scatter^high^ subsets compared with total blasts. Standardised relative fluorescence intensity values (SRFI) were used. (E) Flow cytometric distributions of p-rpS6 for three patient samples. The histograms illustrate fluorescence signals from test antibody (shaded) compared to isotype control (unshaded).

8/13 samples had sufficient CD34^+^CD38^-^ cells (primitive subset) for analysis. The CD34^+^CD38^-^ subset had higher p-rpS6 than the total blast population (Fig 4, P = 0.02). A statistically significant difference in p-mTOR was not observed. MTOR activation features in the maturation and differentiation of normal myeloid cells [8]. Maturing leukaemia cells have an increased cytoplasmic complexity which is shown flow cytometrically as increased side scatter, and furthermore CD45 expression increases with monocytoid maturation [34–36]. To examine mTOR activation in maturing cells, we re-gated our samples on a subset with increased side scatter and high CD45 (illustrated in Fig 1D as region 4). 6/13 samples, all with M4 or M5 FAB types, had distinct CD45^high^ SSC^high^ subpopulations. MTOR and RpS6 were more highly phosphorylated in this subset (P = 0.05, 0.03 respectively).

### 5. 4E-BP1 phosphorylation is decreased in Ki-67¯ and CD34+CD38¯ subsets, contrasting with S6 phosphorylation

**Fig 5:**
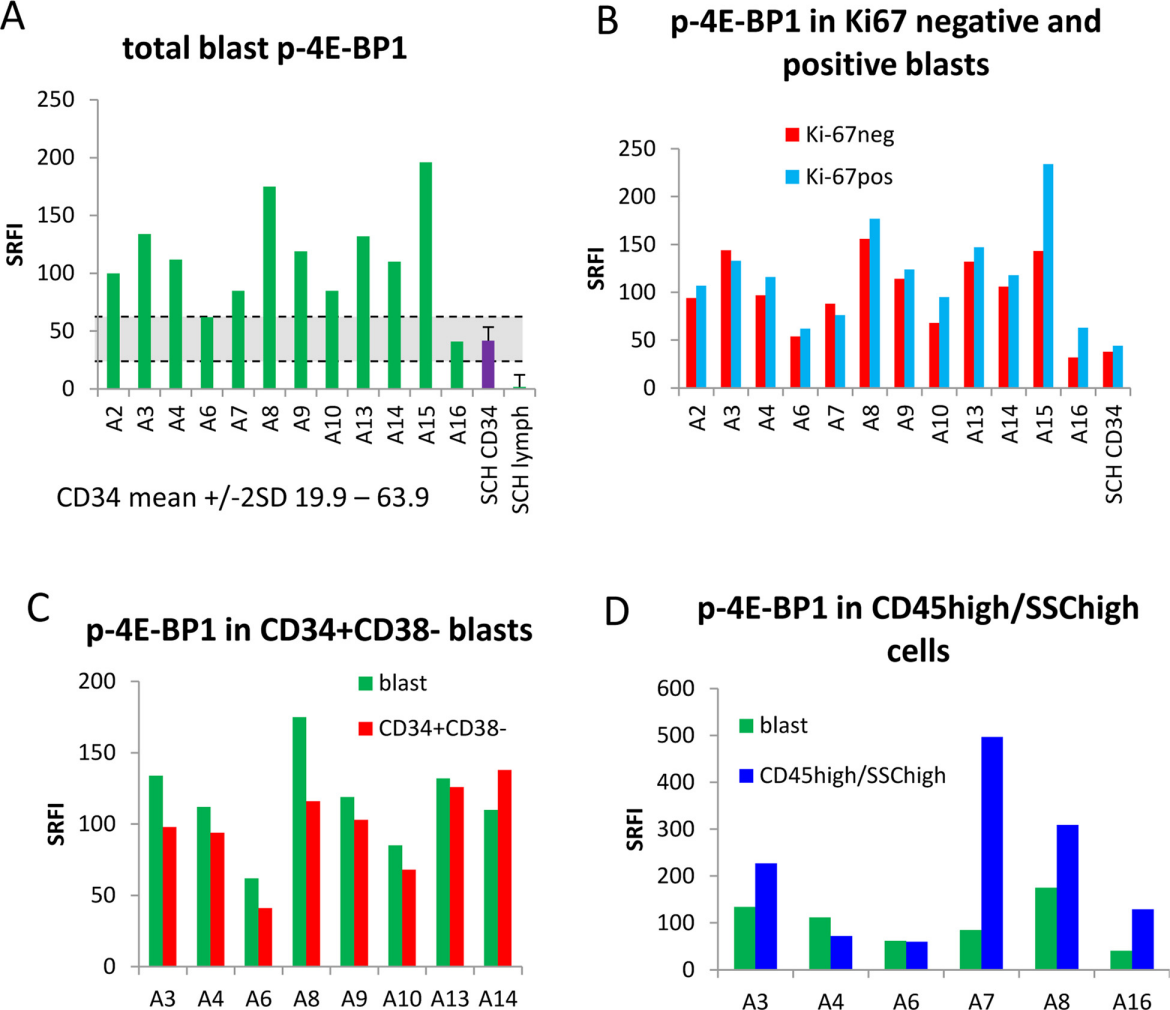
p-4E-BP1 distribution. Summary histograms of p-4E-BP1 show standardised relative fluorescence intensity unit values (SRFI) for (A) blasts from 12 individual primary AML samples and four (averaged) stem cell harvests, (B) Ki-67¯ and Ki67^+^ blast subsets (C) CD34^+^CD38^-^ subsets and (D) CD45^high^/side scatter^high^ subsets compared with total blasts.

4-EBP1 thr36/45 phosphorylation is mediated directly by mTOR [25] and is thus an additional indicator of mTOR activity. We evaluated a directly conjugated antibody to this epitope and were able to document reproducible measurements in SCH CD34+ cells. P-4E-BP1 was documented in these mobilised, normal CD34+ cells but not lymphocytes from stem cell harvests (Fig 5A). When compared to the normal CD34+ cells, 4E-BP1 was hyper-phosphorylated a median of 2.6-fold in AML blasts (Fig 5A). (To allow comparison, we calculated that the median increase in p-rpS6 was 22-fold). In contrast to rpS6, 4E-BP1 was slightly, but significantly, hyper-phosphorylated in the proliferative (Ki-67+) blast subset (by a median of 15%, P = 0.01, Fig 5B) and its expression in the CD34+CD38- subset was only higher than that of bulk blasts in 1/8 samples examined (Fig 5C). (To allow comparison, we calculated that p-4E-BP1 was decreased by a median of 18% in the CD34+CD38- subset compared with total blasts, but p-rp-S6 was elevated by a median 95%). 4E-BP1 was more highly phosphorylated in the CD45^high^ SSC^high^ subpopulations in 4/6 samples (Fig 5D).

## Discussion

In this study, we have measured the phosphorylation status of mTOR pathway molecules in leukaemia cells in conjunction with their proliferation status. CD34^+^ and CD2^+^ cells from mobilised SCH provided normal controls. Absence of Ki-67 has long been used to distinguish cells that have exited from the cell cycle, and indeed the CD2^+^ lymphocytes had the expected Ki-67¯ phenotype [17] with negligible p-mTOR, 4E-BP1 or p-rpS6. In contrast, the normal SCH CD34^+^ cells expressed phosphorylated mTOR and 4E-BP1. These mobilised SCH CD34^+^ cells were mostly (70%) Ki-67^+^, showing that the majority of these were cycling, and we demonstrated in Figs 3 and 5 that p-mTORser2448 and p-4E-BP1thr36/thr45, biomarkers of mTORC1 activation, were no higher in the cycling than the dormant fraction of these normal cells. As mTOR signalling is a central mediator of metabolic activity, driving energy generation, macromolecule synthesis and cell growth [6–8], our results suggest that metabolic activity can be as marked in Ki-67¯ as in Ki-67^+^ cells. Likewise, AML samples failed to show upregulation of mTOR and rpS6 phosphorylations in the Ki-67^+^ subset. Only 4E-BP1 phosphorylation was higher in the the Ki-67^+^ subset, in keeping with its specialised role in proliferation [27], but the increase, although statistically significant, was a modest 15%. On balance, the data indicate that Ki-67¯ mobilised, normal CD34+ cells as well as Ki-67¯ leukaemic blasts are metabolically active. Dormant cells do not all behave in the same way: genes activated and repressed vary with the length of time since the last mitosis and the nature of the quiescence-inducing stimulus [39]. Moreover, dormant short-term haematopoietic stem cells (HSCs) express higher CDK6 than long-term HSCs [40]. It is highly probable that normal, mobilised CD34^+^ cells as well as leukaemia cells, even where exit from cycle is indicated by Ki-67 or CD71 negativity, tend to cycle much more frequently than unstimulated lymphocytes, and maybe this is associated with the ability to maintain a high level of metabolic priming. Dormant AML cells selected by absence of Hoechst33342 and pyronin Y staining have been found to re-enter the cell cycle without exogenous stimulation [14]. Our data suggest that phenotypically dormant leukaemia cells would not be resistant to metabolic targeting such as the synthetic lethal co-activation of AMPK and mTORC1 recently described in AML [41].

Our second unexpected finding was that, when compared to normal CD34^+^ cells from stem cell harvests, p-mTOR was low, 4E-BP1 was 2.6-fold elevated in AML blasts, and p-rpS6 was increased by 22-fold in the blasts, with an additional 95% elevation in leukaemic CD34^+^CD38^-^ cells. What could account for this discordance, especially taking into account that rpS6 phosphorylation is downstream of mTOR activation, as indicated by rapamycin inhibition in Fig 1? Differential phosphorylation of rpS6 and 4E-BP1 by mTORC1 is well-described in the literature, particularly with regard to the variable rapamycin sensitivity of 4E-BP1, which may be attributable to the influence of PIM kinases [5, 42–44]. Differences in total rpS6 expression levels between the cell types are likely to contribute to the high phosphorylation of this protein that we have documented in AML blasts, as shown in S1 Fig. Differences in subcellular localisation may be a contributory factor, as may be relative expression and activation of endogenous inhibitors of rpS6 phosphorylation such as protein phosphatase 1, or activators such as p70S6 kinases and casein kinase [45–48]. RpS6 has additional binding partners which may be involved in its regulation, including the protective chaperone HSP90 [49]. Examination of the 200 AML patient mRNAs in the Cancer Genome Atlas database revealed a close correlation between rpS6 and HSP90 at the level of mRNA expression in primary samples (dataset available from cbioportal.org) [50, 51].

The issue of whether malignant myeloid cells might be more dependent on rpS6 activity than normal dividing CD34+ cells is also raised by our findings. RpS6 phosphorylation-mutant mice are characterised by small cell size, decreased ribosomal biogenesis and muscle weakness, associated with low ATP levels despite normal mitochondrial oxygen consumption [52–54]. The mice have low protein levels of mitochondrial enzymes NDUFS4, and UQCRH [53]. The possible relevance of this latter observation to AML is supported by our examination of the TCGA database, in which these enzymes correlate with rpS6 at the mRNA expression level (S1 Table). The expectation of aerobic glycolysis in all cancer cells has been unravelling over the past few years [3], and leukaemia initiating cells have been shown to use oxidative phosphorylation [55]. By stimulating synthesis of respiratory chain enzymes, rpS6 may contribute to this.

MTOR activity is essential for normal multi-lineage haematopoiesis [56]. The finding that both mTOR and rpS6 are highly phosphorylated in monocytoid AML cells supports a previous finding of high p-rpS6 in these cells [23]. The literature on this topic is complex: whilst mTOR inhibition has been shown to induce differentiation in myeloid leukaemic cell lines, possibly through prolonging PU1 effects, it has also been shown to preserve an undifferentiated phenotype and long term survival in AML cells *in vivo* and *in vitro* [28]. It would also seem logical for differentiated cells to require mTOR pathway activation to meet the metabolic needs of specialisation, and in this respect the monocytoid cells would appear to follow a normal maturation pattern. This contrasts with the hyper-phosphorylation of rpS6, but not of mTOR, in AML blasts, especially in CD34^+^CD38¯ cells. Our data indicate that differences between cell subsets need to be taken into account in predicting and monitoring cellular responses to therapeutics.

A couple of technical discussion points may be useful to some. MTOR pathway phosphorylation is highly dependent on the environment, and our protocol included a 90 minute pre-incubation in serum-containing medium. We abandoned initial protocols of positive selection for CD34^+^ cells from stem cell harvests and of culture in media without serum, as these diminished the signal. The decision to use fluorescence intensity rather than percentage of positive cells was taken based on observations such as those illustrated in Fig 4E, where the whole population’s fluorescence may shift compared to the isotype control, but a highly fluorescent subpopulation may also be seen. Mean fluorescence changes better encompass these two types of change than percentage positivity.

In conclusion, our results indicate that biomarkers of metabolic activity are highly expressed in the Ki-67¯ subsets of both normal SCH CD34^+^ cells and AML blasts. Moreover, although rpS6 ser235/236 phosphorylation is commonly thought of as a surrogate for upstream mTOR activity, our results suggest a hyper-activation of p-rpS6 in the malignant cells only that raises new questions. Firstly, is the hyper-phosphorylation of rpS6 simply an epiphenomenon of oncogene-driven activity, or is it metabolically important (and thus potentially druggable)? Secondly, given the strength of the p-rpS6 signal in AML cells, is dephosphorylation a suitable predictive biomarker for therapeutic responses? AML modalities, such as DNA damaging agents and receptor kinase inhibitors, converge to dephosphorylate rpS6 through their actions on the akt/mTOR, ATM/AMPK/mTOR and ERK pathways, suggesting that further research in this area could be fruitful.

## Supporting Information

S1 Table RPS6 correlations from the Cancer Genome Atlas AML samples. RpS6 phosphorylation is specifically associated with expression of NDUFS4, and UQCRH respiratory chain genes *in vivo* [53]. MTOR activation is associated with transcriptional activation of a wide range of genes, notably HIF1, PFPK and PDK1 (glycolysis), G6PD and RPIA (pentose phosphate pathway), MVK and SC5D (lipid and sterol biosynthesis, see additional reference). We determined the relationship between mRNA expression levels of rpS6 and these genes in the TCGA data of 200 AML samples. Only NDUFS4, and UQCRH were in the top 1% of genes associated with RPS6 (datasets available via cbioportal.org, refs [50, 51].
(DOCX)

S1 Fig Total rpS6 expression is higher in AML samples than stem cell harvests. Total rpS6 was measured using Alexa 488-conjugated antibody (#5317, Cell Signalling Technologies) in CD45/sidescatter blast-gated AML samples and CD45/CD34/sidescatter gated stem cell harvests. Standardisation was with fluorescent beads as described. For AML blasts n = 4, median rpS6 = 178 standardised relative fluorescence intensity (SFRI) units. For n = 3 SCH samples, CD34+ and SSClowCD45high (lymphocyte-gated) median values were 32 SRFI units and 20 SRFI units respectively.
(TIF)
