# Supplementary material for: Phenotypically Dormant and Immature Leukaemia Cells Display Increased Ribosomal Protein S6 Phosphorylation
Source: PLoS One. 2016 Mar 17;11(3):e0151480. doi: 10.1371/journal.pone.0151480 (PMC4795744; doi:10.1371/journal.pone.0151480)
Supplement: S1 Table — RpS6 phosphorylation is specifically associated with expression of NDUFS4, and UQCRH respiratory chain genes in vivo [53]. MTOR activation is associated with transcriptional activation of a wide range of genes, notably HIF1, PFPK and PDK1 (glycolysis), G6PD and RPIA (pentose phosphate pathway), MVK and SC5D (lipid and sterol biosynthesis, see additional reference). We determined the relationship between mRNA expression levels of rpS6 and these genes in the TCGA data of 200 AML samples. Only NDUFS4, and UQCRH were in the top 1% of genes associated with RPS6 (datasets available via cbioportal.org, refs [50, 51]. (DOCX) [file pone.0151480.s001.docx]

**S1 Table. RPS6 correlations from the Cancer Genome Atlas AML samples**

| GENE | Rho value |
| --- | --- |
| NDUFS4 | 0.58 |
| UQCRH | 0.56 |
|  |  |
| RPIA | 0.38 |
| MVK | 0.08 |
| PFKP | 0.06 |
| PDK1 | 0.01 |
| SC5D | -0.03 |
| G6PD | -0.21 |
| HIF1A | -0.24 |

Additional reference:  Duvel K, Yecies JL, Menon S, Raman P, Lipovsky AI, Souza AL, et al. Activation of a metabolic gene regulatory network downstream of mTOR complex 1. Molecular cell. 2010;39(2):171-83. doi: 10.1016/j.molcel.2010.06.022. PubMed PMID: 20670887; PubMed Central PMCID: PMC2946786.
