# Supplementary figures and images for: Phenotypically Dormant and Immature Leukaemia Cells Display Increased Ribosomal Protein S6 Phosphorylation
Source: PLoS One. 2016 Mar 17;11(3):e0151480. doi: 10.1371/journal.pone.0151480 (PMC4795744; doi:10.1371/journal.pone.0151480)

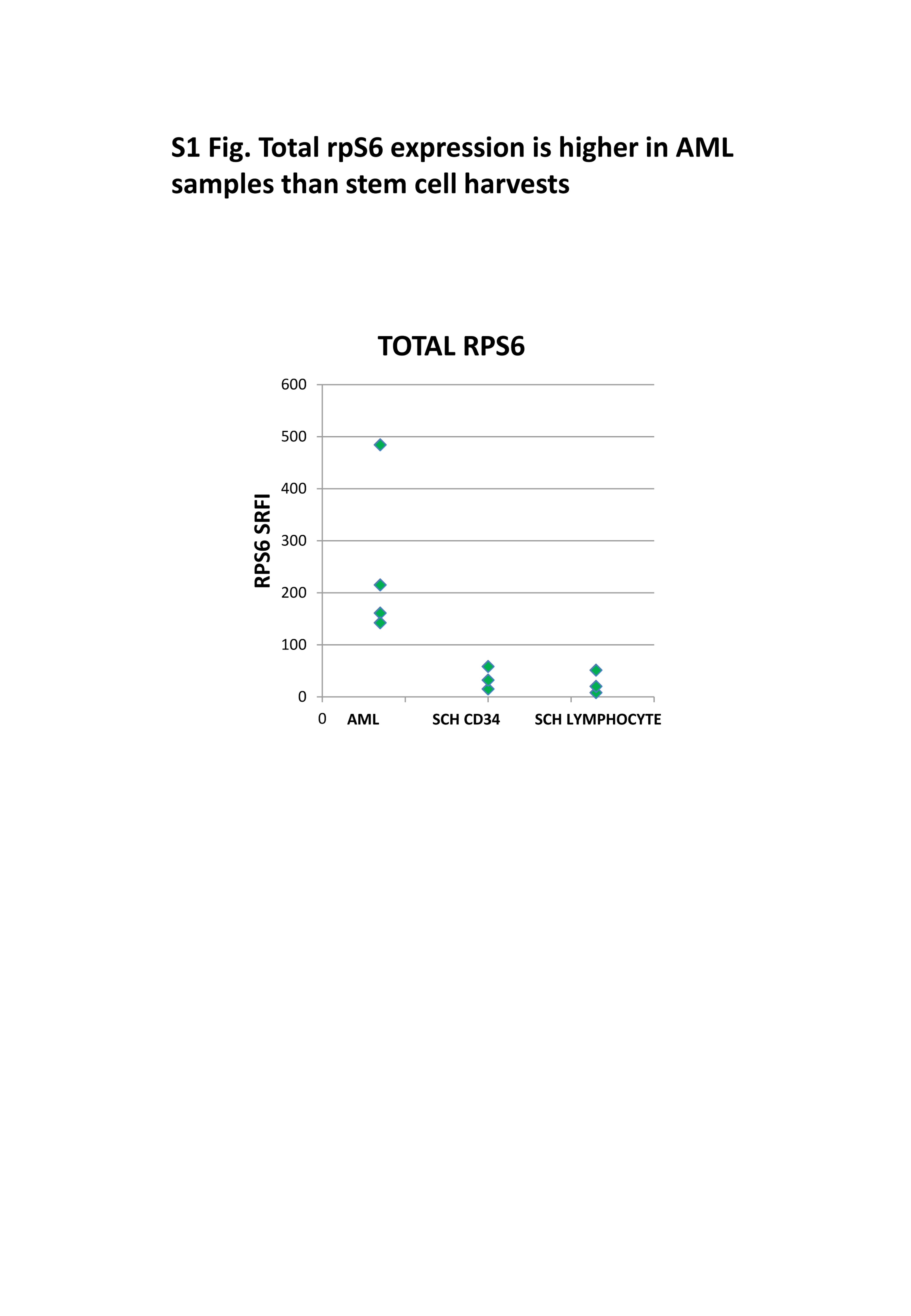

Supplement: S1 Fig — Total rpS6 was measured using Alexa 488-conjugated antibody (#5317, Cell Signalling Technologies) in CD45/sidescatter blast-gated AML samples and CD45/CD34/sidescatter gated stem cell harvests. Standardisation was with fluorescent beads as described. For AML blasts n = 4, median rpS6 = 178 standardised relative fluorescence intensity (SFRI) units. For n = 3 SCH samples, CD34+ and SSClowCD45high (lymphocyte-gated) median values were 32 SRFI units and 20 SRFI units respectively. (TIF) [file pone.0151480.s002.tif]
